# The ColRS system is essential for the hunger response of glucose-growing *Pseudomonas putida*

**DOI:** 10.1186/1471-2180-11-170

**Published:** 2011-07-26

**Authors:** Marta Putrinš, Andres Ainelo, Heili Ilves, Rita Hõrak

**Affiliations:** 1 Institute of Molecular and Cell Biology, University of Tartu, Riia 23, Tartu, Estonia

## Abstract

**Background:**

The survival of bacteria largely depends on signaling systems that coordinate cell responses to environmental cues. Previous studies on the two-component ColRS signal system in *Pseudomonas putida *revealed a peculiar subpopulation lysis phenotype of *colR *mutant that grows on solid glucose medium. Here, we aimed to clarify the reasons for the lysis of bacteria.

**Results:**

We present evidence that the lysis defect of *P. putida colR *mutant is linked to hunger response. A subpopulation prone to lysis was located in the periphery of bacterial cultures growing on solid medium. Cell lysis was observed in glucose-limiting, but not in glucose-rich conditions. Furthermore, lysis was also alleviated by exhaustion of glucose from the medium which was evidenced by a lower lysis of central cells compared to peripheral ones. Thus, lysis takes place at a certain glucose concentration range that most probably provides bacteria a hunger signal. An analysis of membrane protein pattern revealed several hunger-induced changes in the bacterial outer membrane: at glucose limitation the amount of OprB1 channel protein was significantly increased whereas that of OprE was decreased. Hunger-induced up-regulation of OprB1 correlated in space and time with the lysis of the *colR *mutant, indicating that hunger response is detrimental to the *colR-*deficient bacteria. The amount of OprB1 is controlled post-transcriptionally and derepression of OprB1 in glucose-limiting medium depends at least partly on the carbon catabolite regulator protein Crc. The essentiality of ColR in hunger response can be bypassed by reducing the amount of certain outer membrane proteins. In addition to depletion of OprB1, the lysis defect of *colR *mutant can be suppressed by the down-regulation of OprF levels and the hindering of SecB-dependent protein secretion.

**Conclusions:**

We show that *Pseudomonas putida *growing on solid glucose medium adapts to glucose limitation through up-regulation of the sugar channel protein OprB1 that probably allows enhanced acquisition of a limiting nutrient. However, to survive such hunger response bacteria need signalling by the ColRS system. Hence, the ColRS system should be considered a safety factor in hunger response that ensures the welfare of the cell membrane during the increased expression of certain membrane proteins.

## Background

Most bacteria live in constantly changing and often nutritionally limiting environments. The success of bacteria in such conditions depends on their ability to sense the nutritional status of the environment and respond appropriately by reprogramming their gene expression and cell metabolism. For instance, nutrient depletion triggers starvation response that involves the stress-specific sigma factor RpoS and results in drastic changes in gene expression and finally arrests cell growth and division [1]. Bacteria can also discriminate between nutrient-rich and nutrient-poor conditions and respond to nutrient limitation through a regulated nutrient-specific hunger response [2]. Hunger response, activated when the growth rate of a bacterial population decreases due to limited acquisition of nutrients, essentially differs from the starvation response. While the starvation response prepares a cell population for survival in a nutrient-depleted environment, the hunger response improves the ability of bacteria to grow under nutrient-poor conditions [3]. The most obvious bacterial physiological response to low nutrient levels is the enhancement of scavenging ability for the limiting nutrient [2,4]. For instance, if *E. coli *is cultivated in glucose-limited chemostat, its permeability to glucose is increased through up-regulation of several outer membrane porins and high-affinity cytoplasmic membrane transporters [5-8]. However, as the *rpoS *gene was not induced in these conditions, hunger-induced changes should be considered distinct from stationary phase response [8]. Importantly, the mutants that are defective in some hunger-induced transporter have reduced fitness in nutrient-poor conditions [5,9].

Hunger response has been studied by cultivation of bacteria in chemostat which allows a long-term and almost steady-state growth in nutrient-limiting conditions [2]. However, liquid batch cultures of bacteria also transiently experience a nutrient-limited period just before the exhaustion of the carbon source from the medium. Bacteria that grow on solid surfaces, e.g. on agar plates, encounter specific complications of nutrient acquisition, as during consumption of growth substrates niches with different nutrient level develop, which in turn results in a cellular differentiation and an increase in population heterogeneity [10]. The main difference between growth conditions of bacteria in liquid and on solid media is the development of nutrient concentration gradients during the growth on solid medium. This may significantly influence bacterial responses, as has been illustrated by the spatially and temporally different expression of a reporter gene in *Bacillus subtilis *[11,12]. Similarly, nutrient gradients that develop in other types of structured multicellular bacterial consortia, e.g. in biofilms, cause considerable physiological heterogeneity [13]. For example, the *P. aeruginosa *biofilm was reported to contain subpopulations with different metabolic activity: metabolically active bacteria were found in the upper layer and cells with low metabolic activity in the interior of the biofilm. Interestingly, such metabolic heterogeneity resulted in different adaptation responses as well as varied tolerance to antibiotics among subpopulations [14]. Thus, nutrient gradients strongly affect the behaviour of bacterial population on solid media.

*Pseudomonas putida *is a metabolically versatile bacterium widely distributed in the nature [15,16]. The comparison of genomes of *P. putida *and other *Pseudomonas *bacteria revealed 3,708 shared coding sequences [17]. The genes of the ColRS two-component signal transduction pathway are highly conserved in all *Pseudomonas *species [18] and growing evidence shows that the absence of the ColRS two-component system leads to several defects in different pseudomonads. Deficiency in the ColRS system results in the lowered root colonization ability of *P. fluorescens *[19,20] and the attenuated virulence of *P. aeruginosa *[21]. Several ColRS-deficiency related phenotypes are also reported for *P. putida*, including down-regulation of stationary phase mutational processes [22], lowered phenol tolerance [23] and an increased susceptibility of cells to divalent metal ions [24]. We observed recently that under certain circumstances, the ColRS system is essential for the viability of *P. putida*. The *colR*-deficient *P. putida *displays a serious defect on the solid glucose medium where a subpopulation of bacteria lyses as evidenced by the release of cytoplasmic proteins and chromosomal DNA [25]. Intriguingly, the lysis of *colR *mutant occurs only on glucose and not on any other carbon source. Flow cytometry of propidium iodide-stained cells showed that even though most of the glucose-grown *colR*-deficient cells were indistinguishable from the wild-type, a minor subpopulation of cells had a seriously damaged membrane permeable to propidium iodide [25].

In the current study we took different approaches to understand i) why only a subpopulation of *colR *mutant lyses and ii) why the cell lysis occurs only on glucose medium. We identified several mutations that suppressed the lysis phenotype of *colR*-deficient bacteria and indicated that lysis is caused by hunger-induced changes in the outer membrane composition, including the accumulation of sugar channel protein OprB1. We showed that the degree of hunger response and the lysis of bacteria depend on glucose gradient building up in solid medium during the growth of bacteria - both traits were significantly elevated within the peripheral subpopulation of the *colR*-deficient strain. We conclude that ColRS system is needed for the proper response of bacteria to glucose limitation and contributes to the maintenance of membrane homeostasis under the increased expression of nutrient scavenging systems.

## Methods

### Bacterial strains, plasmids, and media

The bacterial strains and plasmids we used are described in Table 1. All experiments were conducted with derivatives of *P. putida *strain PaW85 [26] which is isogenic to fully sequenced KT2440 [27]. Bacteria were grown on Luria-Bertani (LB) medium [28] or on minimal medium [29] containing either 0.2% glucose, 0.2% Na-benzoate or 0.2% gluconate. Some experiments were performed with bacteria grown on media with glucose concentrations of 0.4 and 0.8%. To enhance the lysis of the *colR *mutant, in some experiments 1 mM phenol was added into the solid minimal medium. Congo Red at 0.0005% was added to the medium for visual evaluation of cell lysis. When selection was necessary, the growth medium was supplemented with ampicillin (100 μg/ml), streptomycin (20 μg/ml) or gentamicin (10 μg/ml) for *E. coli *and with carbenicillin (1500 μg/ml), kanamycin (50 μg/ml), streptomycin (300 μg/ml), tetracycline (20 μg/ml) or gentamicin (10 μg/ml) for *P. putida*. *P. putida *was incubated at 30°C and *E. coli *at 37°C. Bacteria were electrotransformed following Sharma and Schimke [30].

**Table 1:** Bacterial strains and plasmids

Strain or plasmid	Genotype or construction	Source or reference
***E. coli***		

CC118 λ*pir*	Δ(*ara-leu*) *araD *Δ*lacX74 galE galK phoA20 thi-1 rpsE rpoB argE*(Am) *recA1 *λ*pir *phage lysogen	[64]

***P. putida***		

PaW85	Wild-type, isogenic to KT2440	[26]

PaWcolR	PaW85 *colR*::Km^r^	[22]

PaWoprB1	PaW85 *oprB1*::Sm^r^	[23]

PaWcolR-oprB1	PaWcolR *oprB1*::Sm^r^	[23]

PaWoprB1-tacB1	PaWoprB1 + *oprB1 *under the control of *tac *promoter and *lacI*^q ^repressor (Sm^r ^Gm^r^)	This study

PaWcolR-oprB1-tacB1	PaWcolR-oprB1 + *oprB1 *under the control of *tac *promoter and *lacI*^q ^repressor (Sm^r ^Gm^r^)	This study

PaWcrc	PaW85 *crc*::Tet^r^	This study

PaWoprB1-tacB1-crc	PaWoprB1-tacB1 *crc*::Tet^r ^(Sm^r ^Gm^r ^Tet^r^)	This study

**Plasmids**		

mTn5SSgusA40	Delivery plasmid for mini Tn5 Sm (Ap^r ^Sm^r^)	[65]

pRK2013	Helper plasmid for conjugal transfer of mTn5SSgusA40 (Km^r^)	[66]

pKTlacZS/C	Promoter probe plasmid pKTlacZ containing *tnpA *promoter of Tn*4652 *fused with *lacZ*	[35]

p9TT_B_lacZ	Promoter probe plasmid (Cm^r ^Ap^r^)	[23]

p9TT1015	p9TT_B_lacZ containing *gtsA *promoter fused with *lacZ *(Cm^r ^Ap^r^)	This study

pBRlacItac	Expression vector containing P_tac _promoter and *lacI*^q ^repressor in pBR322 (Ap^r^)	[67]

pBRlacItac/oprB1	pBRlacItac containing *oprB1 *as a HindIII-XbaI fragment under the P_tac _promoter (Ap^r^)	This study

pUCNotKm	pUC18Not derivative with Km^r ^gene instead of Ap^r ^(Km^r^)	R. Teras

pUCNotKm/tacoprB1	pUC18NotKm containing BamHI fragment with *lacI*^q^-P_tac_-*oprB1 *cassette (Km^r^)	This study

pBK-miniTn7-ΩGm	pUC19-based delivery plasmid for miniTn7-ΩGm (Ap^r ^Gm^r^)	[68]

pminiTn7Gm/tacoprB1	pBK-miniTn7-ΩGm containing NotI fragment with *lacI*^q^-P_tac_-*oprB1 *cassette (Ap^r ^Gm^r^)	This study

pCRC10	pKNG101 containing *sucB *and *crc *interrupted with tetracycline resistance gene (Sm^r ^Tet^r^)	[32]

### Selection of the suppressors of the lysis of the *colR*-deficient *P. putida*

For the identification of genes implicated in cell lysis, the *colR*-deficient strain was subjected to mutagenesis using a Tn*5 *based mini-transposon that contains a streptomycin resistance marker. Mini-transposon-carrying plasmid mTn5SSgusA40 was conjugatively transferred from *E. coli *CC118 λ*pir *into *P. putida colR*-deficient strain with the aid of the helper plasmid pRK2013. Transconjugants with random chromosomal insertions of the mini-transposon were selected on 0.2% glucose minimal plates supplemented with kanamycin, streptomycin, Congo Red and 1 mM phenol. We searched for white colonies amongst the pink ones. Screening of about 28,000 transposon insertion derivatives of the *colR*-deficient strain disclosed 25 clones with significantly reduced Congo Red staining. To identify chromosomal loci interrupted in these clones, arbitrary PCR and sequencing were used. PCR products were generated by two rounds of amplification as described elsewhere [31]. In the first round, a primer specific for the Sm gene (Smsaba - 5'-GAAGTAATCGCAACATCCGC-3') and an arbitrary primer (Arb6 - 5'-GGCCACGCGTCGACTAGTACNNNNNNNNNNACGCC-3') were used. Second-round PCR was performed with the primers SmSplopp (5'-GCTGATCCGGTGGATGACCT-3') and Arb2 (5'-GGCCACGCGTCGACTAGTAC-3').

### Cloning procedures and the construction of bacterial strains

For the overexpression of OprB1 in the *oprB1 *and *colRoprB1 *strains, the PCR-amplified *oprB1 *gene was first cloned under the control of the *tac *promoter and *lacI*^*q *^repressor in pBRlacItac. *oprB1 *was amplified from *P. putida *PaW85 genome using oligonucleotides oprB1ees (5'-GGCAAGCTTCAAAGGCCGTTGACTCG) and oprB1lopp (5'-TGGTCTAGAGCTCTTGTTGTTTGAGAT) complementary to the upstream and downstream regions of the *oprB1 *gene, respectively. PCR product was cleaved with HindIII and XbaI and inserted into pBRlacItac opened with the same restrictases. The *lacI*^q^-P_tac_-*oprB1 *cassette was excised from pBRlacItac/oprB1 with BamHI and subcloned into BamHI-opened pUCNotKm resulting in pUCNotKm/tacoprB1. Finally, the *oprB1 *expression cassette was inserted as a NotI fragment into the gentamicin resistance-encoding minitransposon in the delivery vector pBK-miniTn7-ΩGm yielding pminiTn7Gm/tacoprB1. To introduce the *oprB1 *expression cassette into the chromosome of *P. putida *PaW*oprB1 *or PaW*colR-oprB1*, we performed triparental mating between *P. putida *strain, *E. coli *CC118 λ *pir *carrying pminiTn7Gm/tacoprB1, and a helper plasmid pRK2013-containing *E. coli *HB101. Transconjugants were selected on minimal plates that contained gentamicin and streptomycin. The chromosomal presence of the *lacI-*P_tac_*-oprB1 *cassette of transconjugants was verified by PCR and inducible expression of OprB1 was proved by the OM protein analysis.

To disrupt the *crc *gene, the plasmid pCRC10 was employed [32]. By using triparental mating this plasmid was transferred into *P. putida *wild-type strain PaW85 as well as into OprB1 over-expression strain PaW*oprB1-tacB1*. Transconjugants were first selected on tetracycline and streptomycin-containing benzoate minimal plates. Secondary screen was performed on LB plates supplemented with 10% sucrose. Sucrose-resistant colonies were picked up and the disruption of *crc *was verified by PCR using the primers PPcrcall (5'-ATCGCTACCCGATGATCTGG) and PPcrcylem (5'-TCTTGCTATCGACGATGGCG).

To make the transcriptional fusion of *gtsA *(PP1015) with *lacZ *reporter gene, we used the promoter probe plasmid p9TT_B_lacZ. The 980-bp-long *gtsA *promoter region was amplified from *P. putida *PaW85 chromosome using oligonucleotides PP1014kesk (5'-GCTGTCGACGCCAATACGCT) and PP1015alg (5'-GCATCTAGACGAAGCGTGGAATTCATC). The PCR-amplified DNA fragment was cleaved with HincII and XbaI and ligated into SmaI-XbaI-opened p9TT_B_lacZ, yielding p9TT1015.

### β-galactosidase assay

β-galactosidase activities were measured either from solid or liquid medium-grown bacteria. For the analysis of *gtsA *promoter, total enzyme activity was measured using permeabilized cells as described elsewhere [33].

### Cell lysis assay

To evaluate the cell lysis of the *colR *mutant, we have previously used so-called unmasked β-galactosidase assay which relies on the detection of a cytoplasmic enzyme β-galactosidase leaked out from the cells [25,34]. In this assay we measured the β-galactosidase activity in suspension of cells permeabilized with SDS and chloroform (total activity), and also in intact, non-permeabilized cells. The percentage of unmasked β-galactosidase activity was calculated from equation: xn/xp × 100%, where xp is β-galactosidase activity measured in SDS and chloroform-treated cells, and xn is β-galactosidase activity measured in non-permeabilized cells. We have shown earlier that in case of ColR-deficiency-dependent cell lysis, unmasked β-galactosidase values are above 5% [25]. As a source of β-galactosidase, the plasmid pKTlacZS/C containing the *lacZ *gene, was used [35]. Bacteria were grown for 24, 48, or 72 hours on glucose (0.2, 0.4, or 0.8%) or gluconate (0.2%) M9 minimal media. To enhance lysis, 1 mM phenol was added to the growth medium in some experiments. Bacteria were scraped off the agar plate using plastic swabs and suspended in M9 solution. Optical density of the cell suspension was determined at 580 nm and β-galactosidase activity was measured [34].

### Isolation of outer membrane proteins

For the isolation of outer membrane proteins (OMPs) bacteria were grown for 24 hours on two Petri plates. Bacteria were scraped off the agar and suspended in 3 ml of 10 mM HEPES buffer (pH 7.4). For the analyses of peripheral and central subpopulations, bacteria were grown on agar plate in sectors as pictured in Results. To collect enough cells from the sectors, five to ten plates were used, i.e., cells from 15 to 30 sectors per strain were collected and suspended in 3 ml of 10 mM HEPES buffer (pH 7.4). Cells were disrupted by ultrasonication and the cell debris was pelleted by centrifugation at 10 000 g at 4°C for 10 minutes. The supernatant was then centrifuged at 100 000 g at 4°C for 1 hour to pellet membrane proteins. In order to dissolve inner membrane proteins the pellet was resuspended in 2 ml of 10 mM HEPES buffer containing 1% of N-lauroylsarcosine (sodium salt) and incubated at 37°C for 30 minutes. Samples were then centrifuged at 100 000 g at 4°C for 1 hour and the pellet containing OMPs was washed with 3 ml of 10 mM HEPES buffer. After final centrifugation at 100 000 g at 4°C for 1 hour the pellet was suspended in 100 μl of 10 mM HEPES buffer. Protein concentration was measured using the Bradford assay. Two to four independent OMP preparations were made from each strain grown in particular conditions.

### Identification of OprE by LC-MS/MS analysis

OM proteins were resolved by SDS-PAGE and visualized by Coomassie Blue staining. The band of interest was excised from the gel and in-gel digested with modified sequencing grade trypsin (Promega), as in [36]. Peptides from in-gel-digested samples were purified with StageTips [37] and analyzed by LC-MS/MS using an Agilent 1200 series nanoflow system (Agilent Technologies, Santa Clara, CA) connected to a LTQ Orbitrap classic mass spectrometer (Thermo Electron, Bremen, Germany) that was equipped with a nanoelectrospray ion source (Proxeon, Odense, Denmark). Up to five data-dependent MS/MS spectra were acquired in centroid in the linear ion trap for each FTMS full-scan spectrum. Fragment MS/MS spectra from raw files were extracted as MSM files and then merged to peak lists by using Raw2MSM version 1.7 [38] selecting the top six peaks for 100 Da. MSM files were searched with the Mascot 2.3 search engine (Matrix Science, London, UK) against the protein sequence data base composed of *Pseudomonas putida *KT2440 sequences and common contaminant proteins such as trypsin, keratins, etc.

### Measurement of residual glucose concentration in agar medium

Bacteria were grown in three distantly located sectors on minimal agar medium containing 0.2, 0.4 or 0.8% glucose. After 24, 48, and 72 hours of growth residual glucose concentration in the agar was determined. Using sterile 1-ml pipette tips, small plugs were cut from two regions of the agar plate - just adjacent to the growth area of bacteria and underneath the cells. To excise a plug from underneath the growth area, the cells were first scraped off. Agar plugs were melted at 100°C and cooled to 65°C. Glucose content in melted agar was determined with Glucose Liquicolor kit (Human GmbH, Germany) according to the instructions of the manufacturer.

## Results

### Glucose-specific lysis of the *colR *mutant occurs only on solid medium and increases in time

**Figure 1:**
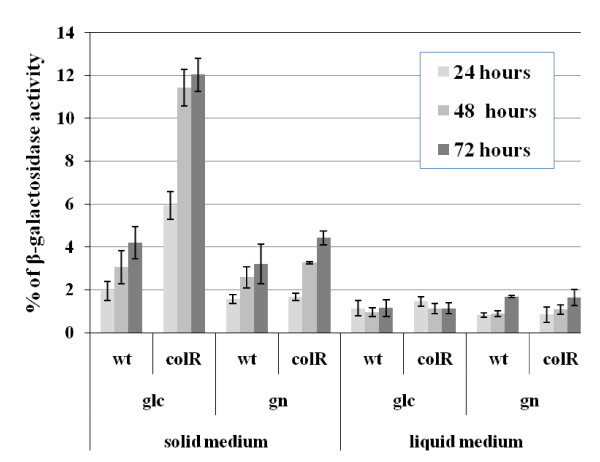
**Unmasked β-galactosidase activity as indicator of cell lysis**. The data present percentage of β-galactosidase activity, measured from non-permeabilized cells against the total β-galactosidase activity determined from permeabilized bacteria. Results for *P. putida *PaW85 (wt) and *colR*-deficient (colR) strains measured at 24, 48 and 72 hours are shown. Bacteria were grown for three days either on solid or in liquid M9 minimal medium with 0.2% glucose (glc) or gluconate (gn) as carbon sources. Data (mean ± standard deviation) of at least three independent determinations are presented.

To specify the requirements for the glucose-related lysis of the *colR*-deficient *P. putida*, cell lysis was measured at different time points of growth both on solid and in liquid media with either glucose or gluconate as a carbon source. Cell lysis was evaluated in previously described assay [25] that measures cytoplasmic β-galactosidase leaked out from the cells (unmasked β-galactosidase activity, see Methods). Absence of ColR resulted in cell lysis only on glucose-containing solid medium and not in the liquid one (Figure 1). We observed an increase of cell lysis of the *colR *mutant in time until 48 hours of growth, and thereafter the unmasked β-galactosidase activity stabilized at the level of about 12% of total β-galactosidase activity (Figure 1). In good accordance with our previous results, *colR*-dependent lysis did not occur on gluconate medium [25]. These data suggest that ColRS system is particularly important for *P. putida *that grows on glucose solid medium.

### Identification of suppressor mutations of glucose-specific lysis of the *colR*-deficient bacteria

**Figure 2:**
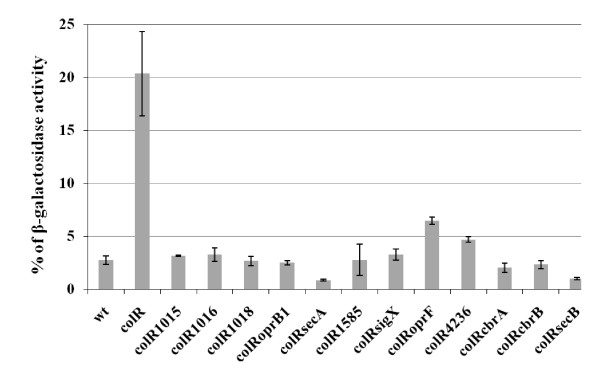
**Unmasked β-galactosidase activity as indicator of cell lysis of Congo Red non-binding derivatives of the *colR*-deficient strain**. The data present percentage of β-galactosidase activity, measured from non-permeabilized cells against the total β-galactosidase activity determined from permeabilized bacteria. Results for *P. putida *PaW85 (wt), *colR*-deficient strain (colR), and for different transposon insertion derivatives of the *colR *mutant are shown. Bacteria were grown for 24 hours on solid 0.2% glucose M9 minimal medium containing 1 mM phenol. Data (mean ± standard deviation) of at least three independent determinations are presented.

In order to identify the genes involved in the glucose-dependent cell lysis, the *colR*-deficient strain was subjected to transposon mutagenesis to isolate suppressor mutations. In this screen the ability of the *colR *mutant colonies to bind Congo Red was used as a marker for lysis phenotype [25]: white transconjugants were searched among pinkish *colR *mutant colonies. As cell lysis and Congo Red binding of *colR-*deficient bacteria are significantly enhanced in the presence of phenol [25], suppressor screen was performed on glucose minimal plates supplemented with Congo Red and 1 mM phenol. Analysis of about 28,000 transposon insertion derivatives of the *colR*-deficient strain disclosed 25 clones with significantly reduced Congo Red staining. Sequencing of mini-transposon insertions revealed 12 different suppressor genes, and most of these were picked up more than once (Table 2). The isolated white clones were tested in respect to cell lysis by using unmasked β-galactosidase assay. Data in Figure 2 show that all isolated clones not binding Congo Red also had significantly lower unmasked β-galactosidase activity compared to the parental *colR*-deficient strain, and most of them behaved exactly like the wild type. Thus, the results of β-galactosidase assay show a clear correlation between Congo Red binding ability and cell lysis confirming that the identified genes are indeed implicated in the glucose-specific lysis of the *colR *mutant.

**Table 2:** List of transposon insertion derivatives of the *colR *mutant with reduced Congo Red staining

Locus ID	Gene	Product name	Number of clones (disruption site)*	Probable localization#
PP1015	*gtsA*	sugar ABC transporter, periplasmic sugar binding protein	2 (269; 587)	P

PP1016	*gtsB*	sugar ABC transporter, permease protein	2 (717; 972)	CM

PP1018	*gtsD*	sugar ABC transporter, ATP-binding protein	2 (414; 1094)	CM

PP1019	*oprB1*	porin B1	3 (66; 146; 909)	OM

PP1345	*secA*	preprotein translocase subunit SecA	2 (2 × 2695)	C

PP1585		antidote protein, putative	1 (203)	unknown

PP2088	*sigX*	extracytoplasmic function sigma factor SigX	4 (251; 304; 336; 480)	C

PP2089	*oprF*	porin F	1 (849)	OM

PP4236	*dsbE*	thiol:disulfide interchange protein DsbE	1 (526)	CM

PP4695	*cbrA*	sensor histidine kinase CbrA	4 (2 × 10; 936; 1763)	CM

PP4696	*cbrB*	response regulator CbrB	1 (1013)	C

PP5053	*secB*	preprotein translocase subunit SecB	2 (9; 314)	C

* The numbers in the brackets denote nucleotides in the coding sequence of particular gene just before the minitransposon insertion.

# Abbreviations: CM - cytoplasmic membrane, OM - outer membrane, C - cytoplasm, P - periplasm

Inspection of identified genes (Table 2) revealed that in accordance with our previous results [25], disruption of the *oprB1 *(PP1019) gene did eliminate the lysis. Knockouts of sugar transport genes located upstream of *oprB1*, i.e., *gtsA *(PP1015), *gtsB *(PP1016), and *gtsD *(PP1018) also suppressed the lysis phenotype of the *colR *mutant. In addition to sugar transport genes, lysis was also suppressed by inactivation of the two-component system CbrA-CbrB, which is known to regulate several catabolic pathways and the cellular ratio of carbon to nitrogen [39,40]. The death of the *colR *mutant was also prevented by the knockout of a sigma factor SigX, which regulates expression of major outer membrane protein OprF in *Pseudomonas aeruginosa *and *Pseudomonas fluorescens *[41]. Consistent with that, inactivation of *oprF *also suppressed lysis of the *colR *mutant. It is noteworthy that the disruption of the SecA and SecB components of the general Sec protein secretion pathway also eliminated the lysis (Table 2). The isolation of a *secA*-knockout in our screen was particularly surprising because SecA has been shown essential not only for Sec pathway but also for the viability of bacteria [42]. Sequencing of two independently identified *secA *mutants revealed that they both possessed minitransposon insertion at the very end of the *secA *gene - between 37 and 38 nt from the stop codon (Table 2). Therefore, these mutants most probably coded for a truncated SecA protein lacking the last 12-13 amino acids. The most distal C-terminal part of SecA is necessary for its binding with SecB, a chaperone which keeps precursor proteins in translocation-competent state and targets them to SecA [42]. Literature data shows that although SecA is essential for bacteria, its SecB-binding domain is dispensable for protein secretion and cell viability [43,44]. Thus, we consider that the *secA *mutants that were picked up in our suppressor screen are impaired only in SecB-dependent protein secretion and in respect of the cell lysis phenotype they resemble *secB*-knockouts. Finally, unique insertions of transposon into PP1585 and PP4236, coding for putative antidote protein of a toxin-antitoxin system and a thiol:disulfide interchange protein, respectively, also resulted in white non-lysing colonies of the *colR *mutant. In conclusion, inactivation of different genes prevented lysis of the *colR *mutant and most of these genes encode either membrane proteins or are implicated in regulating membrane proteins.

### Analysis of the outer membrane composition of the non-lysing transposon derivatives of the *colR *mutant

**Figure 3:**
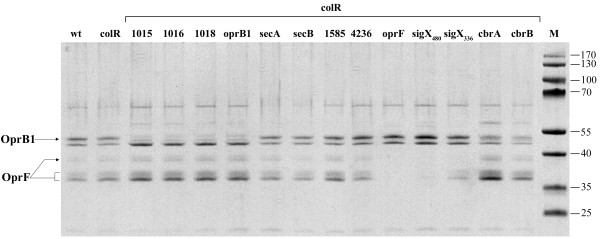
**SDS-PAGE of outer membrane protein preparations stained with Coomassie Blue**. OM proteins were extracted from 24-hour-old populations of bacteria grown on solid minimal medium with 0.2% glucose. Representative results of the *P. putida *PaW85 (wt), *colR*-deficient (colR), and of different transposon insertion derivatives of the *colR*-deficient strains are shown. Arrows indicate locations of the channel proteins OprB1 and OprF (calculated molecular weights 49.6 kD and 37 kD, respectively). All lanes contain 0.5 μg of OM proteins.

The results of the suppressor analysis predict that the *colR *mutant cannot maintain membrane protein homeostasis. This is supported by two phenomena. First, the reduction of protein secretion by the inactivation of the SecB-dependent protein export suppresses cell lysis. Second, the disruption of genes for the outer membrane porins, OprB1 and OprF, also eliminated the lysis indicating that the outer membrane (OM) composition may be unbalanced in the *colR*-deficient *P. putida*. In order to address this issue we compared the pattern of OM proteins of the wild-type and the *colR *mutant as well as the suppression mutants of the *colR *strain. Data in Figure 3 demonstrate that the overall OM protein pattern of the wild-type and the *colR *strains is similar. The PP1585, PP4236, *secA *and *secB *derivatives of the *colR *mutant also have OM protein profiles that are quite similar to the wild-type. However, as expected, OM protein preparations of the *colRoprB1 *and *colRoprF *mutants respectively lacked OprB1 and OprF channel proteins. Note that OprF is represented by several differently migrating forms. This is consistent with previous data on several OM porins, including OprF of *P. aeruginosa*, showing that these proteins are prone to modification by heat and β-mercaptoethanol treatment that is carried out for the solubilization of proteins before applying to the gel [45]. Given that *sigX *and *oprF *genes comprise one operon and that OprF is positively regulated by SigX in *P. aeruginosa *and *P. fluorescens *[41], it was expected that all four different *colRsigX *knockout strains have significantly lowered OprF amount in their OM (Figure 3, only two *colRsigX *derivatives are presented). However, while three *sigX *derivatives of the *colR *mutant (minitransposon insertions after nucleotides 251, 304 and 336 of the *sigX *gene) revealed only modestly reduced expression of OprF (Figure 3, only *colRsigX*_336 _is presented), the *colRsigX *strain with most distal transposon insertion in *sigX*, displayed drastically decreased OprF level (Figure 3, see *colRsigX*_480_). An analysis of the sugar transport gene knockouts (PP1015, PP1016 and PP1018) showed that they all possessed a significantly decreased amount of OprB1. Thus, disruption of the genes located upstream of *oprB1 *seems to have a polar effect on the OprB1 expression. Actually, this is in good agreement with recent results reporting that sugar transport genes comprise one transcriptional unit with *oprB1 *[46]. OM fractions of *colRcbrA *and *colRcbrB *mutants were generally similar to the wild-type and the *colR *mutant, but still had slightly less OprB1 protein than the parental strain. Thus, OM analysis shows that although the pattern of OM proteins of the *colR *mutant resembles that of the wild-type, its defects can be suppressed by decreasing the amount of OprB1 or OprF in OM.

### Overexpression of OprB1 induces cell lysis, especially in the *colR*-deficient background

**Figure 4:**
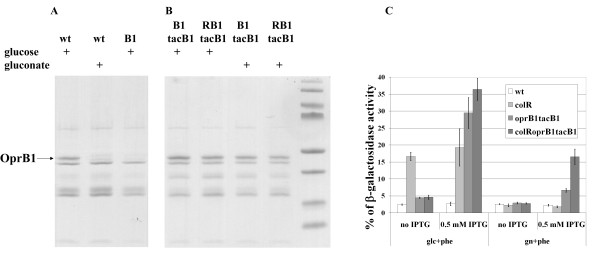
**Effect of the OprB1 overexpression on the profile of outer membrane proteins and cell lysis**. A and B. SDS-PAGE of outer membrane protein preparations stained with Coomassie Blue. Representative results of the *P. putida *PaW85 (wt), *oprB1*-deficient (B1) as well as OprB1-overexpressing strains PaW*oprB1-tacB1 *(B1tacB1) and PaW*colR-oprB1-tacB1 *(RB1tacB1) are presented. OM proteins were extracted from 24-hour-old populations of bacteria grown on solid minimal medium with either 0.2% glucose or gluconate. OM proteins presented in panel B have been purified from the cells which were grown in the presence of 0.5 mM IPTG. Plus (+) marks above the lanes designate a particular carbon source added to the growth medium. Arrow indicates location of OprB1. C. Quantification of cell lysis by the unmasked β-galactosidase assay. Bacteria were grown for 24 hours on solid 0.2% glucose (glc) or 0.2% gluconate (gn) minimal medium containing 1 mM phenol (+phe). For the induction of OprB1 0.5 mM IPTG was used. Data (mean ± standard deviation) of at least three independent determinations are presented.

The analysis of the OM protein pattern of transposon mutants suggested that the *colR*-deficient *P. putida *cannot tolerate the natural load of membrane proteins, at least that of OprB1 and OprF when growing on glucose solid medium. Here, it is important to note that the *colR *mutant is prone to lysis specifically on glucose but not on gluconate [25] despite both these substrates are degraded through Entner-Doudoroff pathway. While most of the genes for glucose and gluconate metabolism are induced by both these carbon sources, one of them, *oprB1*, is specifically expressed only during glucose growth [46,47]. Our results also show that OprB1, a major OM protein in glucose-grown cells, is not detectable in gluconate-grown *P. putida *(Figure 4A). Therefore, we hypothesized that the glucose-induced expression of OprB1 could be the major determinant of glucose-specific cell lysis of the *colR*-deficient bacteria. If so, then artificial overexpression of OprB1 should result in the cell lysis of the *colR *mutant on both the glucose and the gluconate medium. To test this assumption, we introduced an extra copy of the *oprB1 *gene under control of IPTG-inducible *tac *promoter to the *oprB1-*deficient strains PaW*oprB1 *and PaW*colR-oprB1*. The *oprB1*-deficient background was used to avoid an unequal amount of OprB1 in glucose and gluconate growing cells due to glucose-specific induction of the native *oprB1 *locus. The OM analysis of PaW*oprB1-tacB1 *and PaW*colR-oprB1*-*tacB1 *strains revealed that induction of *tac *promoter with 0.5 mM IPTG resulted in equal OprB1 expression in both strains and in case of both carbon sources (Figure 4B). OprB1 protein was not detected in cells without IPTG-induction (not shown). Unmasked β-galactosidase activity assay demonstrated that overexpression of OprB1 caused the lysis of the *colR *mutant also on the gluconate medium (Figure 4C), which confirms the importance of the amount of OprB1 in OM as a major determinant of cell lysis. Furthermore, even the *colR*-proficient PaW*oprB1-tacB1 *strain did not tolerate the artificial overexpression of OprB1, revealing a clear lysis phenotype on both carbon sources. This data suggests that OM is highly sensitive to the abundance of OprB1 and obviously the natural amount of OprB1 induced by glucose is close to the saturating level that the bacterium can tolerate.

### The degree of lysis of the *colR *mutant depends on the location of cells in the solid medium population and on the glucose concentration in the medium

**Figure 5:**
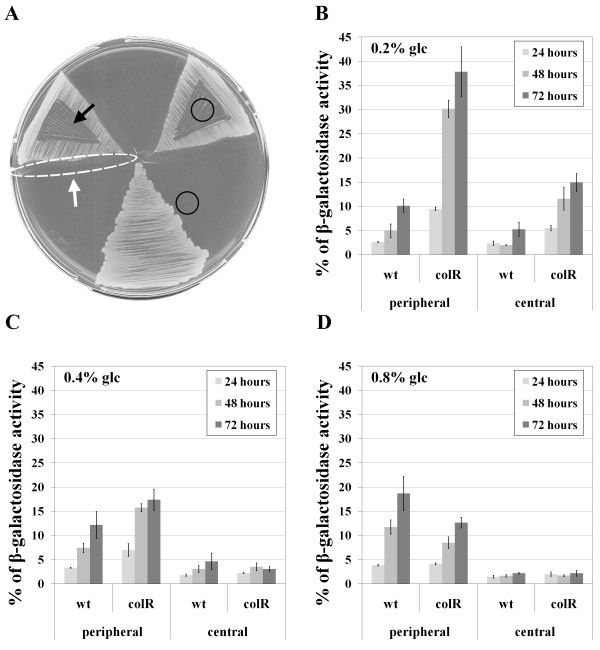
**Comparison of lysis of peripheral and central subpopulations of *P. putida *PaW85 wild-type (wt) and *colR*-deficient (colR) strains grown on solid glucose medium**. A. Representation of a Petri plate with three growth sectors of bacteria and subpopulations sampled for β-galactosidase analysis. Unmasked β-galactosidase activity was assayed from the cells of peripheral subpopulation (area encircled by the dotted line and indicated by the white arrow) and from central one (indicated by the black arrow). Black circles indicate the areas sampled for the measurement of residual glucose concentration in the medium (data is presented in Table 3). The degree of lysis is presented as unmasked β-galactosidase activity which was measured from bacteria grown either 24, 48 or 72 hours on solid media with 0.2% (B), 0.4% (C) or 0.8% (D) of glucose (glc) as the carbon source.

Two remarkable features of the glucose-specific cell lysis of the *colR*-deficient strain are that it can be observed only on solid medium (Figure 1) and that only a fraction of population lyses [25] indicating heterogeneity among the bacteria. Therefore we decided to test the effect of the location of cells in a population on their lysis. For that, the *colR*-deficient bacteria were grown on agar plates with 0.2% glucose and lysis was analysed in cells withdrawn from two different regions of bacterial lawn on agar plate sectors - the periphery and the centre. Bacteria were streaked as shown in Figure 5A to enhance the build-up of nutrient gradients. Unmasked β-galactosidase activity measured at 24, 48 and 72 hours of growth clearly indicated that at every time-point the lysis of *colR *mutant was always significantly higher among peripheral cells of the bacterial lawn compared to the central subpopulation (Figure 5B). Also wild-type bacteria revealed spatially different unmasked β-galactosidase activity demonstrating up to two-fold higher enzyme values at 48 and 72 hours in case of peripheral cells (Figure 5B).

Due to the spatiotemporal character of the lysis of the *colR *mutant we hypothesized that nutrient limitation could be involved in cell death. During the active growth of bacteria on agar plate the concentration of glucose in the growth area decreases, yet, it is obvious that compared to the central population the peripheral cells are nutritionally less limited due to diffusion of glucose from the adjacent medium. To evaluate the glucose consumption dynamics during 72 hours of bacterial growth on 0.2% (9 mM) glucose solid medium, we measured the glucose concentration in the growth agar by sampling the regions underneath the cell lawn and adjacent to the bacterial growth area (sampling regions are indicated in Figure 5A). Already at 24 hours of growth, the amount of glucose in the medium underneath the bacterial lawn had dropped below the detection level of the assay (0.1 mM). Concentration of glucose in the medium adjacent to the growth area continuously dropped down to 1.6 mM by 72 hours of growth (Table 3). These results show that bacteria constantly consume glucose that is diffusing from adjacent region of agar plate and that peripheral population of bacteria has to adapt to gradient of glucose. Notably, glucose consumption dynamics for the wild-type and the *colR *mutant were similar.

**Table 3:** Glucose concentration in the bacteria-free agar medium adjacent to the growth area of the cells

Glucose concentration (mM)			
**Initially**	**After 24 hours**	**After 48 hours**	**After 72 hours**

9 (0.2%)	6.9 ± 0.3	2.9 ± 0.6	1.6 ± 0.2

18 (0.4%)	14.0 ± 1.0	5.9 ± 0.4	3.5 ± 0.4

36 (0.8%)	29.2 ± 0.3	13.0 ± 1.3	6.8 ± 0.9

Accumulating evidence indicates that bacteria growing under subsaturating nutrient levels express a transient response called hunger response, which helps them to cope with limiting conditions [48]. The most obvious feature of hunger response is up-regulation of nutrient uptake systems, including several OM porins [3,5]. This lead us to hypothesize that elevated lysis of peripheral cells on 0.2% glucose plates resulted from the hunger response of bacteria while central cells were already starving at that time and therefore showed only minimal hunger response. If so, then the lysis of peripheral cells should be suppressed by increasing the glucose concentration in the medium. Thus, we assessed the cell lysis of peripheral and central subpopulations under different glucose concentrations. Data in Figure 5C and 5D clearly shows that the lysis of the *colR*-deficient strain inversely correlates with the glucose concentration in the medium. While the increase of the initial glucose concentration in the medium up to 0.4% (two-fold) had no effect on the unmasked β-galactosidase activity of the wild-type (compare Figure 5B and 5C), in *colR*-deficient background this increase significantly reduced the lysis of peripheral cells and eliminated the lysis of central cells (Figure 5C). If the growth medium of bacteria contained 0.8% of glucose instead of 0.2%, then both peripheral and central subpopulations of *colR *mutant behaved similarly to the wild-type, i.e., showed no ColR-depletion-dependent lysis (Figure 5D). In a parallel experiment we also monitored the glucose concentration in the agar plate and observed that after 24 hours of growth the glucose was already exhausted (residual concentration below 0.1 mM) from underneath the cell lawn even if the initial glucose concentration in the medium was 0.4 or 0.8%. At the same time, the glucose concentration in the adjacent medium was relatively high although it was constantly decreasing over time (Table 3). There was an inverse correlation between the lysis of peripheral cells of *colR*-mutant and glucose concentration adjacent to the growth area - irrespective of the initial glucose concentration (0.2, 0.4, or 0.8%), the lower the glucose concentration in adjacent region was, the greater was the lysis (Table 3 and Figure 5). If initial glucose concentration in the medium was 0.8%, it did not decrease below 6 mM in the region adjacent to the cell growth area during the experiment (Table 3). This level is obviously too high to initiate the lysis of the *colR*-deficient strain. This data strongly suggests that particularly the hungry fraction of the *colR *mutant is liable to lysis.

### Amount of OprB1 in OM inversely depends on glucose concentration

**Figure 6:**
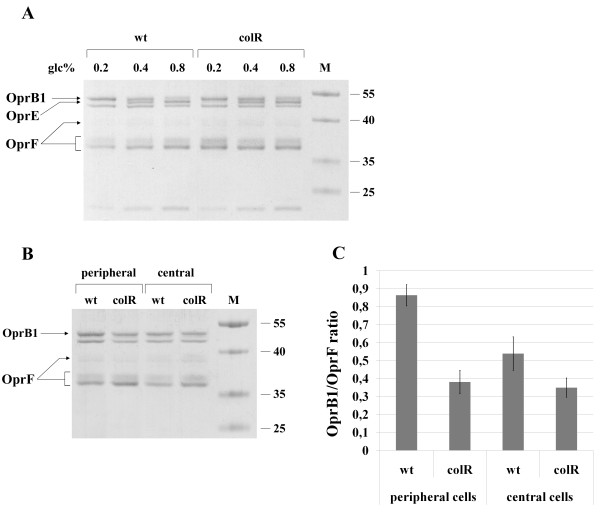
**Profiles of the outer membrane proteins of the *P. putida *PaW85 (wt) and the *colR*-deficient (colR) strains under different growth conditions**. OM proteins were purified from the solid medium-grown *P. putida *PaW85 (wt) and *colR*-deficient (colR) strains cultivated on the agar plate sectors as illustrated in Figure 5A. A. OM protein profiles of 24-hour-old peripheral subpopulations of bacteria grown on solid medium with 0.2, 0.4 or 0.8% glucose. Location of OprB1, OprE, and OprF is indicated by the arrows. B. OM protein profiles of peripheral and central subpopulations grown for 24 hours on 0.2% glucose solid medium. The quantified protein bands are indicated by the arrows. C. The ratio of OprB1 to OprF in different subpopulations of the *P. putida *wild-type and the *colR *mutant strains grown for 24 hours on 0.2% glucose solid medium. The OprB1/OprF ratio was calculated from the data obtained from at least two independent protein preparations and from three independent gel runs. Mean values and 95% confidence intervals are presented.

After establishing conditions which enhance (peripheral growth) and diminish (higher glucose concentration) the lysis of *colR *mutant cells, we asked whether we can see some changes in the OMP composition under respective conditions. As the abundance of OprB1 in OM was promoting cell lysis, we hypothesised that the level of OprB1 may inversely depend on glucose concentration. To test that, we analysed the pattern of OM proteins of the wild-type and the *colR*-deficient bacteria grown on agar plates with different concentrations of glucose. Analysis of OMP fraction purified from peripheral subpopulations revealed a dissimilar OM protein pattern for bacteria grown at different glucose concentrations (Figure 6A). First, the OM preparations of bacteria grown at 0.4 or 0.8% of glucose revealed an additional OM protein (~50 kD) that was barely detectable in the membrane preparations of bacteria grown at 0.2% of glucose. A similar pattern was observed also for the OMP preparation of central cells (data not shown). Mass spectrometric analysis identified this hunger-repressed protein as OprE encoded by PP0234 (Figure 6A). Second, the amount of OprB1 inversely correlated with initial glucose concentration in agar plates being highest at 0.2% and lowest at 0.8% of glucose (Figure 6A). Note that the differences observed for OprB1 amounts in OM correlated well with the lysis data of the *colR *mutant on different glucose plates (Figure 5). All these results support the hypothesis that an elevated expression of OprB1 due to nutrient limitation generates membrane stress that is not tolerated by the *colR *mutant and results in the lysis of most vulnerable subpopulation of bacteria.

When analysing the composition of OM proteins of bacteria grown on 0.2% glucose (conditions that promote lysis), we repeatedly observed a slight difference between the wild-type and the *colR *mutant regarding the relative proportions of OprB1 and OprF. The *colR *mutant showed a tendency to have less OprB1 and more OprF in OM than the wild-type. This was most clearly seen when the OM protein profiles of peripheral subpopulations of two strains were compared (for representative results see Figure 6B). In order to quantify the proportions of OprB1 and OprF in the OMP preparations, we analysed the SDS-PAGE images with ImageQuant TL program. Quantification showed that OM of the wild-type indeed contained relatively more OprB1 than that of the *colR*-deficient strain (Figure 6C, p = 8,6e-07 and p = 6,8e-04 for preparations from peripheral and central cells, respectively). Furthermore, the relative amount of OprB1 was higher in peripheral wild-type cells compared to that of central cells (p = 6,2e-05), indicating the elevated hunger response of peripheral cells. Differently from the wild-type, the OprB1/OprF ratio for the peripheral and the central cells of the *colR *mutant was similar. We suggest that the increased level of OprB1 in OM that is normally induced in response to glucose limitation is unbearable to the *colR *mutant and therefore does not rise above a certain threshold level.

### Hunger-induced expression of OprB1 is regulated post-transcriptionally

**Figure 7:**
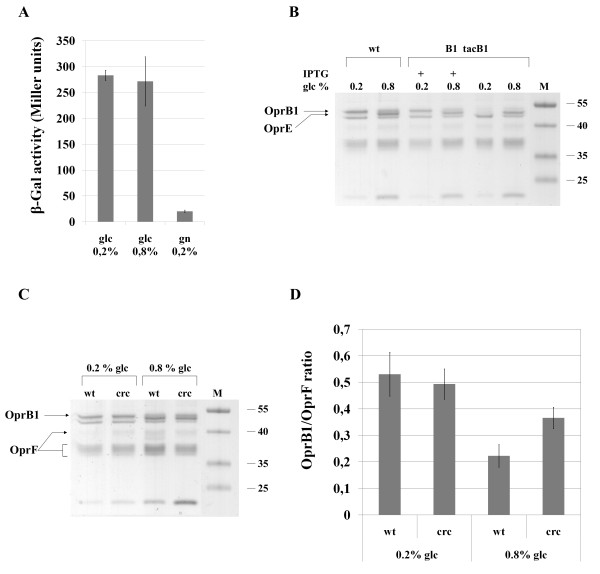
**Post-transcriptional regulation of OprB1 depends on the glucose concentration**. A. β-Galactosidase (β-Gal) activity expressed from the *gtsA *promoter was measured in the wild-type *P. putida *grown on solid medium with 0.2 or 0.8% glucose or 0.2% gluconate. B. SDS-PAGE of the outer membrane protein preparations from *P. putida *wild-type PaW85 (wt) and from OprB1-overexpressing strain PaW*oprB1-tacB1 *(B1tacB1) grown 24 hours over the whole Petri plate. The growth medium contained 0.2 or 0.8% glucose (glc) as a carbon source. Plus (+) mark above the lane indicates that the bacterial growth medium contained also 0.5 mM IPTG. C and D. Analysis of the effect of the *crc *inactivation on the hunger-induced up-regulation of OprB1. The outer membrane proteins were prepared from *P. putida *wild-type (wt) and *crc *mutant strains (crc) grown for 24 hours as a lawn over the entire Petri plate. The growth medium contained 0.2 or 0.8% glucose (glc). The ratio of OprB1 to OprF was calculated from the data of at least two independent protein preparations and five independent gel runs. Mean values and 95% confidence intervals are presented.

To test the possibility that expression of OprB1 under glucose limitation increases due to enhanced transcription of glucose transport operon (genes *gtsA *to *oprB1*), the transcriptional fusion of *gtsA *with *lacZ *reporter was constructed and analysed under different glucose concentrations. Results in Figure 7A show that the expression of the *gtsA *promoter is induced by glucose regardless of its concentration. This was also confirmed in the liquid glucose medium by β-galactosidase measurements throughout the growth (data not shown). To find out whether OprB1 expression may be regulated post-transcriptionally we employed the PaW*oprB1-tacB1 *and PaW*colR-oprB1-tacB1 *strains with *oprB1 *gene under the control of IPTG-inducible *tac *promoter. We presumed that if the expression of OprB1 is post-transcriptionally suppressed at high glucose and, *vice versa*, derepressed under glucose limitation, then it should not be possible to artificially overexpress OprB1 from *tac *promoter in glucose-rich environment, i.e., on 0.8% glucose medium. As predicted, the *tac *promoter-originated artificial expression of OprB1 was lower at 0.8% glucose compared to that at 0.2% glucose (Figure 7B). As a matter of fact, it did not exceed the amount of OprB1 characteristic for the wild-type cells growing on glucose-rich medium. This data strongly suggests that hunger-dependent regulation of OprB1 occurs post-transcriptionally. Here, it is relevant to remind that the amount of OprB1 is slightly reduced in *cbrA *and *cbrB *mutants (Figure 3) suggesting that the CbrA-CbrB system is involved in the OprB1 regulation. Recently, CbrA-CbrB system has been shown to act as a positive regulator of CrcZ which is an antagonist sRNA of catabolite repression control protein Crc [49]. The RNA-binding Crc is a global translational regulator of catabolite repression in pseudomonads [50-52]. Interestingly, if *P. putida *grows on amino acid-rich LB medium, the glucose transport genes are repressed by Crc [53]. Furthermore, sequences similar to Crc binding consensus were found in the proximity of the AUG start site of *gtsA *and *oprB1 *genes [50]. The Crc protein therefore seemed to be a likely candidate for translational repression of OprB1 in the glucose-rich solid medium. Thus, we constructed the *crc*-deficient strains and analyzed the effect of Crc inactivation on the amount of OprB1 in OM under glucose-rich (0.8%) and glucose-limiting (0.2%) conditions. Data in Figure 7 (panels C and D) show that Crc indeed affects the relative abundance of OprB1 in OM; yet, this was observed only under glucose-rich conditions in which *crc *mutant displayed higher OprB1/OprF ratio than the wild-type (p = 8.6e-05). However, although inactivation of *crc *alleviated repression of OprB1 on 0.8% glucose medium, the OprB1/OprF ratio was still higher on 0.2% glucose medium (Figure 7D, compare results for the *crc *mutant on 0.2 and 0.8% glucose, p = 6.7e-04). Therefore we conclude that in addition to the Crc some other factor(s) as yet unknown should be implicated in hunger-induced up-regulation of OprB1.

## Discussion

Previous studies on ColRS signaling system have revealed a peculiar subpopulation lysis phenotype of the *colR *mutant grown on glucose solid medium [25]. In this study we clarified the reasons for glucose-specific cell lysis and revealed that the ColRS system is necessary for *P. putida *to survive the hunger response which includes up-regulation of sugar channel OprB1.

Several lines of evidence obtained in this study suggest that the glucose-growing *colR *mutant experiences envelope stress caused by the accumulation of membrane proteins. This was first indicated by the collection of mutants suppressing the lysis phenotype of the *colR*-deficient strain. These data demonstrated that the loss of ColR can be suppressed by down-regulation of certain OM proteins like OprB1 and OprF, as well by hindering the SecB-dependent protein secretion. Second, artificial overexpression of sugar channel protein OprB1 further highlighted the specifically increased sensitivity of the *colR *mutant to this particular OM protein. Although neither the wild-type nor the *colR*-deficient strain tolerated the elevated expression of OprB1 exhibiting lysis phenotype on both glucose and gluconate medium, the *colR *mutant was remarkably more affected (Figure 4C). Third, the *colR*-deficient strain possessed slightly less OprB1 in its OM than the wild-type (Figure 6C), indicating that the membrane of the *colR *mutant is probably sensitive to accumulation of OprB1. Thus, our data suggest that ColRS is necessary for *P. putida *to maintain the cell membrane homeostasis and this becomes particularly important during up-regulation of certain OMPs such as OprB1.

We detected the glucose-specific cell lysis of the *colR*-deficient strain only on solid and not in liquid medium (Figure 1). Bacterial population growing on solid medium is highly heterogeneous and it is obvious that bacteria located at the edge of the growth area experience different conditions compared to the cells in the centre of the population. Gradient fields of carbon source as well as of excreted metabolites develop during the growth, putting the cells in the centre of the population under more restrictive conditions than those at the periphery. It has been shown that such gradient fields govern cellular responses of multicellular solid medium populations and regulate development of gene expression patterns in space and time [11]. Our previous results revealed a spatial aspect of ColR-dependent lysis. Colonies of the *colR*-deficient strain developed central concavities when growing on the glucose medium which we interpreted as an elevated lysis of central population [25]. Here, we proved that the degree of lysis of the *colR *mutant is spatially different. However, contrary to our expectations the lysis of peripheral cells was significantly higher than that of the central cells. Yet, it is important to point out that in the current study we analyzed the bacteria grown on a sector (1/6 of the Petri plate), the area of which is more than 100 times bigger than that of a single colony. Therefore, the nutrient gradients building up in the medium under central cells of a sector and under the central part of a colony are not really comparable. We suggest that lysis occurs at a certain glucose concentration range and whether this develops in the centre or in the periphery of a population depends on the size of the cells' growth area.

This study indicated that the glucose-specific lysis of the *colR*-deficient *P. putida *occurs among a subpopulation of cells adapting to nutrient limitation. This was most strongly evidenced by the fact that the degree of lysis depended both on time and glucose concentration. We suggest that the continuous increase of the *colR *mutant lysis during the first 48 hours of growth on 0.2% glucose solid medium (Figure 1 and Figure 5) is caused by a gradual decrease of glucose concentration. Given that significantly less lysis was observed on 0.4% glucose and that no lysis was detected on 0.8% glucose medium (Figure 5), it is possible to conclude that the ColR-dependent cell lysis occurs only when the amount of glucose decreases below a certain threshold level. However, cell lysis was alleviated also at the exhaustion of glucose which was evidenced by lesser lysis of the central cells compared to the peripheral cells (Figure 4). These data suggest that glucose exhaustion itself is not a trigger of the *colR *mutant lysis; rather, this mutant cannot respond adequately to a certain glucose concentration range which finally causes cell death. This scenario also allows to explain the absence of the lysis phenotype in liquid glucose medium. Obviously, the period of nutrient limitation is transient in liquid batch culture and could have been easily missed in our experiments. Literature data suggest that bacteria growing under suboptimal levels of nutrient, i.e. under conditions between the feast and the famine, express cellular responses that are significantly different from those of rapid growth and starvation [3,48]. Under conditions of hunger when a nutrient becomes limiting but is not yet depleted, bacteria increase permeability of the membrane to facilitate nutrient entry. For instance, a significantly increased expression of the OprF porin and the LamB-Mgl high-affinity glucose uptake system is considered to be the hunger response of *E. coli *under glucose limitation [5]. Analogously, we detected essential nutrient concentration-dependent changes in the OM protein composition of the glucose-grown *P. putida*. We found that the abundance of the sugar channel OprB1 was significantly increased and that of OprE was drastically decreased under low glucose concentrations (Figure 6A). Interestingly, in addition to being modulated by glucose, the abundance of OprE also responds to anaerobiosis [54,55] suggesting that this outer membrane channel contributes to the adaptation to various environmental conditions. OprB1 is known to mediate high-affinity glucose transport both in *P. putida *and *P. aeruginosa *[25,56,57]. While OprB1 is not essential for the glucose transport at higher substrate concentrations, it becomes rate-limiting in nutrient uptake at micromolar (1-10 μM) glucose concentrations [25,57]. Therefore, the up-regulation of OprB1 at low glucose concentrations can be considered an adaptive response of hungry bacteria to stimulate glucose acquisition. However, our results show that the spatiotemporal expression of OprB1 generates spatiotemporal lethal toxicity for *colR*-deficient bacteria, which implies that the ColRS two-component system is an essential regulator of the hunger response of the glucose-growing *P. putida*.

Our data demonstrate that the up-regulation of OprB1 in response to hunger is controlled post-transcriptionally and that catabolite repression control (CRC) protein Crc is one of the factors involved in this regulation (Figure 7). CRC is an important global control system in bacteria allowing hierarchical assimilation of substrates under simultaneous presence of several possible carbon sources. Interestingly, while in many bacteria glucose is a preferred carbon source, *Pseudomonas *prefers organic acids and amino acids to glucose [51,58]. Recent transcriptomic and proteomic data have revealed that Crc protein inhibits several glucose transport and metabolism genes when *P. putida *grows in nutrient-rich LB medium [53]. For instance, the inactivation of the *crc *gene resulted in three times higher abundance of OprB1 in LB-grown cells [53]. Interestingly, it was recently reported that Crc is not important for the growth of *P. putida *DOT-T1E on glucose as single carbon source and this was explained by dispensability of Crc in the medium lacking nutrients alternative to glucose [52]. However, our data demonstrate that Crc can actually affect the usage of glucose as the sole carbon source because the abundance of OprB1 was shown to be elevated in the *crc *mutant. Yet, the effect of Crc on the amount of OprB1 was observed only in glucose-rich but not in glucose-limiting conditions (Figure 7D) suggesting that the Crc-mediated repression of OprB1 is probably completely absent in hungry bacteria allowing a full expression of OprB1. Thus, in addition to regulating the hierarchical use of carbon sources in complete medium, Crc is also involved in fine tuning single carbon source assimilation.

The up-regulation of the glucose-scavenging OprB1 is the most appropriate behavior of *P. putida *at glucose limitation. However, "there is no free lunch in nature." Data of this study suggest that hunger response is costly and if not regulated properly, it might be even deadly as judged by the requirement of ColRS signaling. Interestingly, a largely similar cell death phenomenon was recently characterized in *E. coli *where constitutive expression of the maltoporin LamB resulted in cell lysis in the absence of a functional response regulator OmpR [59,60]. The authors proposed that cell death resulted from envelope stress involving an imbalance in the lipopolysaccharide/porin composition of the outer membrane and an increased requirement for inorganic phosphate [60]. Analogous scenario can be considered for the *colR *mutant, as recent studies conducted in *P. fluorescens *and *Xanthomonas citri *have indicated that ColRS system is involved in LPS production and/or modification [20,61].

Our current study describes not only the participation of ColRS system in hunger response of *P. putida*, but also provides clues to better understand the role of this system in root colonization. It is notable that the colonization defect observed for *P. fluorescens *ColRS system mutant became evident only under the condition of competition with the wild-type strain [19]. This indicates that the colonization ability *per se *is not impaired but rather some other population-related trait is hampered in the absence of ColRS signaling. Our results suggest that hunger-induced lysis of a subpopulation may be responsible for the reduced fitness of the *colR *mutant under competition conditions. Nutrient concentration in the rhizosphere is low [62] and thereby rhizosphere colonization takes place under condition of hunger [63]. We assume that nutrient limitation may result in the hunger-induced subpopulation lysis of the *colR *mutant and this obviously will reduce the fitness of *colR *mutant bacteria in co-cultivation experiments. However, if the *colR *mutant grows as a pure culture, its colonization ability is not affected because nutrients liberated from lysed cells probably support the growth of surviving population. In the future, it would be very interesting to examine the impact of the ColRS system on the viability of different *Pseudomonas *species in the rhizosphere.

## Conclusions

**Figure 8:**
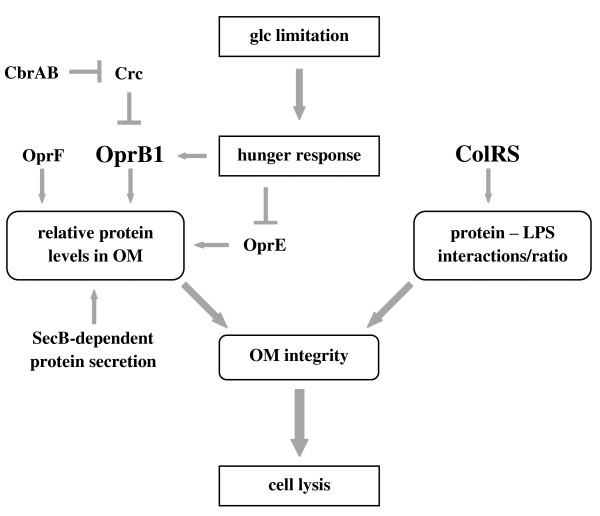
**Schematic representation of factors associated with the glucose concentration-dependent cell lysis of the *colR*-deficient *P. putida***.

Current study demonstrated that the glucose-growing *P. putida *responds to a low glucose level by the up-regulation of the sugar channel OprB1, which most probably facilitates nutrient scavenging under hunger conditions (Figure 8). We present evidence that on the glucose-rich medium the OprB1 expression is post-transcriptionally repressed, and carbon catabolite repression regulator Crc is partially responsible for that. Most interestingly, we show that the hunger-induced expression of OprB1 is lethal to bacteria deficient in ColR as deduced from a clear correlation between the amount of OprB1 and the cell death of the *colR *mutant. However, the glucose-induced death of the *colR *mutant can be suppressed by reducing the abundance of various membrane proteins such as the OprB1 and OprF as well as excluding the SecB-dependent protein secretion (Figure 8). Thus, the ColRS system could be considered a safety factor of hunger response as it ensures the welfare of cell membrane during increased synthesis of certain membrane proteins.

## Authors' contributions

MP and RH prepared design of experimental work. MP carried out transposon mutagenesis screen and participated in OMP analysis. AA purified OMPs and did OMP pattern analysis. HI constructed mutant strains and contributed enzyme assays. RH performed lysis assays, coordinated experimental work and wrote the manuscript. All authors participated in manuscript editing and approved the final manuscript.
